# Recognition of endophytic *Trichoderma* species by leaf-cutting ants and their potential in a Trojan-horse management strategy

**DOI:** 10.1098/rsos.160628

**Published:** 2017-04-05

**Authors:** Silma L. Rocha, Harry C. Evans, Vanessa L. Jorge, Lucimar A. O. Cardoso, Fernanda S. T. Pereira, Fabiano B. Rocha, Robert W. Barreto, Adam G. Hart, Simon L. Elliot

**Affiliations:** 1 Department of Entomology, Universidade Federal de Viçosa, Viçosa, 36570-900 Minas Gerais, Brazil; 2 Department of Phytopathology, Universidade Federal de Viçosa, Viçosa, 36570-900 Minas Gerais, Brazil; 3 Department of Natural and Social Sciences, University of Gloucestershire, The Park, Cheltenham, Gloucestershire GL50 2RH, UK; 4 CAB International, E-UK, Egham, Surrey TW20 9TY, UK

**Keywords:** fungal bodyguards, *Leucoagaricus*, pest management, silviculture, *Trichoderma* endophytes

## Abstract

Interactions between leaf-cutting ants, their fungal symbiont (*Leucoagaricus*) and the endophytic fungi within the vegetation they carry into their colonies are still poorly understood. If endophytes antagonistic to *Leucoagaricus* were found in plant material being carried by these ants, then this might indicate a potential mechanism for plants to defend themselves from leaf-cutter attack. In addition, it could offer possibilities for the management of these important Neotropical pests. Here, we show that, for *Atta sexdens rubropilosa*, there was a significantly greater incidence of *Trichoderma* species in the vegetation removed from the nests—and deposited around the entrances—than in that being transported into the nests. In a no-choice test, *Trichoderma-*infested rice was taken into the nest, with deleterious effects on both the fungal gardens and ant survival. The endophytic ability of selected strains of *Trichoderma* was also confirmed, following their inoculation and subsequent reisolation from seedlings of eucalyptus. These results indicate that endophytic fungi which pose a threat to ant fungal gardens through their antagonistic traits, such as *Trichoderma*, have the potential to act as bodyguards of their plant hosts and thus might be employed in a Trojan-horse strategy to mitigate the negative impact of leaf-cutting ants in both agriculture and silviculture in the Neotropics. We posit that the ants would detect and evict such ‘malign’ endophytes—artificially inoculated into vulnerable crops—during the quality-control process within the nest, and, moreover, that the foraging ants may then be deterred from further harvesting of ‘*Trichoderma*-enriched’ plants.

## 1. Introduction

Symbiotic fungi play a vital role in plant communities. This is well known from mycorrhizal associations which directly influence plant diversity, ecosystem variability and productivity [1,2], as well as plant immunity [3]. However, there is increasing evidence that endophytes may also have a pivotal role in plant-ecosystem functioning [4,5]. Here, we use the term endophyte to refer to microorganisms, in this case fungi, which for all or part of their life cycle live asymptomatically within plant tissues. Although the definition of what constitutes an endophyte has been modified over time [6], our interpretation falls within the currently accepted concept of endophytism which can embrace commensalism, mutualism and parasitism [7].

A second example of symbiosis is that between leaf-cutting ants and the basidiomycete fungus *Leucoagaricus* (Agaricales: Agaricaceae), in which the ants cultivate the fungus in subterranean gardens and harvest the nutrient-rich vegetative bodies (gongylidia) produced by the fungus: a process that has been likened to human agriculture and which evolved around 8–12 Ma [8,9]. Leaf-cutting ants of the genera *Acromyrmex* and *Atta* (Myrmicinae: Attini) are considered to be a keystone group in the natural ecosystems where they occur in the Neotropics—especially in tropical forests—since they affect and generally improve the diversity, productivity and nutrient flow of these biomes [10–12]. However, as highlighted by Fowler *et al*. [10], there is a dilemma when they encroach on human-dominated ecosystems since their impact is always negative, to the point that they can become a major constraint to production in agriculture and silviculture, as well as troublesome in urban situations. Annual losses in Brazilian sugarcane have been put at over 3 tons ha^−1^[13], at a cost of around US$60 million [14], while it has been estimated that a single nest of *Atta* consumes over 1 ton of eucalyptus leaves annually [15], and that, in such plantations, some 30% of management costs are spent on control of leaf-cutting ants [16]. Thus, leaf-cutting ants have become highly problematic in the extensive eucalyptus plantations in the Atlantic forest region of southeast Brazil [17,18]. In Venezuela, *Atta laevigata* at high densities (more than 30 nests ha^−1^) can reduce wood productivity in young plantations of *Pinus caribaea* by up to 50% [19]. These considerations make leaf-cutting ants one of the most important pests of forest plantations in Latin America [16,20]. The issue of leaf-cutting ants as pests is particularly salient as products based on the insecticides sulfluramid and fipronil are currently being phased out from certified usage [21], so there is an urgency to the need for research into understanding these insects and providing alternative means of control. In this context, microbial biological control is a possibility, requiring itself an understanding of how leaf-cutting ants interact with microorganisms.

Here, we investigate the potential of one symbiotic system (plants and fungal endophytes) to influence another (Attini-*Leucoagaricus*). In a previous study, we demonstrated that *Atta laevigata* may exert a form of quality control within the nest and reject leaves containing those fungal endophytes that pose a threat to the colony [22]. Such a threat comes from species of the genus *Trichoderma* (Hypocreales: Hypocreaceae): well-documented antagonistic fungi [23] that are also associated with Attini fungal gardens [22,23], and which can be endophytic in the leaves and stems of tropical trees [24,25]. We found that isolates of this fungus were significantly more common in rejected compared with foraged material [22]. In this study, we consider another leaf-cutting ant, *Atta sexdens rubropilosa* and pose the question: is *Trichoderma* also recognized as a threat to this system? We then discuss how *Trichoderma* might be exploited to manage leaf-cutting ants by incorporating them into vulnerable crops as bodyguards in a Trojan-horse scenario.

## 2. Material and methods

### 2.1. Sampling

Sampling was conducted in two remnant areas of Atlantic forest: a seasonal, subtropical, semi-deciduous, montane forest, ‘Mata do Seu Nico’ (Fazenda Bonsucesso), 20°45′23″ S and 42°52′23″ W, 750 m in altitude; and Estação de Pesquisas, Treinamento e Educação Ambiental Mata do Paraíso (Departamento de Engenharia Florestal, Universidade Federal de Viçosa), 20°48′07″ S and 42°51′31″ W, at a mean altitude of 690 m, both near Viçosa, in the Zona da Mata of Minas Gerais State, southeast Brazil.

Leaf samples were collected from three large colonies of *Atta sexdens rubropilosa* (each nest measuring approx. 25 m^2^ at the surface) at each site. Ten nocturnal collections were made from each nest, at weekly intervals from January to February 2010. Each collection consisted of 50 leaf pieces that were being carried by foraging workers and 50 rejected pieces that were found scattered on the downslope at the side of or below the nest entrances [22]. It is important to note that rejected leaf material showed no evidence of degradation; exhibiting only a colour change from green to chlorotic or brownish-grey (see fig. 1 in [22]). A total of 6000 leaf pieces were collected: 3000 being carried and 3000 that had been rejected from the nest. The material was kept in a refrigerator (approx. 5°C) overnight and processed the following morning.

All material was surface-sterilized as follows: leaf pieces were washed twice in sterile tap water then immersed for 1 min in 70% (v/v) ethanol, followed by 4 min in sodium hypochlorite (5% v/v), and then washed three times in sterile-distilled water (SDW). After surface sterilization, the samples were transferred aseptically to plates containing V8 vegetable broth agar (VBA) [26], supplemented with the antibiotic terramycin (Pfizer) at 1.5 ml/1000 ml. Sealed plates were incubated at room temperature (25 ± 2°C). An initial screening for *Trichoderma* isolates was conducted macroscopically, based on the fast growth rate and green colony coloration typical of the genus. This preliminary identification was subsequently confirmed microscopically based on the characteristic conidiogenesis of *Trichoderma* [27].

The presence of *Trichoderma* was also investigated in the leaves of plants from the foraging area of each nest that had been examined. The collections were made randomly from plants around each of the six marked ant colonies, in the period from February to March 2010. Two old and two new leaves of 20 plants from each area were collected (for a total of 120 plants or 480 leaves). Four segments (5 mm^2^) of each leaf type (new and old) were cut from the middle lamina of each leaf and surface-sterilized, as above. Sterilized leaf pieces were placed on VBA and were incubated at room temperature (25 ± 2°C) for detection of *Trichoderma* isolates, as described above.

### 2.2. Molecular identification of *Trichoderma*

For DNA extraction, mycelia were grown in liquid culture in potato dextrose broth (PDB). The extraction was done with Wizard^®^ Genomic DNA Purification Kit (Promega, USA). The gene Elongation Factor (EF1α) was considered as the species marker [23,28–30].

PCR amplifications were performed using Dreamtaq (Thermo Scientific) and the primers Ef728F (Forward: 5′-CATCGAGAAGTTCGAGAAGG-3′) and Ef986R (Reverse: 5′-TACTTGAAGGAACCCTTACC-3′) following the methodology of Carbone & Kohn [31]. This involved treatment at 94°C for 1 min for initial denaturation, followed by 30 cycles at 94°C for 1 min for denaturation, then at 55°C for 1 min for annealing and 50 s at 74°C for DNA extension, and a final extension at 74°C for 7 min. PCR products were cleaned with ExoSAP-IT^®^ (USB Corporation). The DNA fragment sequences were obtained through the Sanger *et al*. [32] method by Macrogen^®^. The sequences were edited at DNA Dragon (http://www.dna-dragon.com) and deposited on GenBank (http://www.ncbi.nlm.nih.gov). Sequences of EF1α that represent the analysed *Trichoderma* species were obtained from National Center for Biotechnology Information (http://www.ncbi.nlm.nih.gov/).

Sequences were aligned using MAFFT (http://mafft.cbrc.jp/alignment/server/) and adjusted manually using Bioedit (http://www.mbio.ncsu.edu/bioedit/bioedit.html). Alignments are deposited in TreeBASE (http://www.treebase.org/).

All isolates were analysed through Bayesian inference generating a cladistic tree that enabled their identification. The isolates grouped in a species complex clade were re-analysed with the species belonging to the complexes. The best substitution model (GTR + I + G) was obtained by MrModeltest 2.3 [33]. The Bayesian analyses were performed on CIPRES Science Gateway using the tool MrBayes on XSEDE [34] and the following parameters: Lset nst = 2, rates = gamma; Prset statefreqpr = dirichlet (1,1,1,1); mcmc ngen = 10 000 000, samplefreq = 1000, nruns = 2, nchains = 4; sump burnin = 2500, nruns = 2; sumt burnin = 2500, nruns = 2. The Bayesian sampling runs were analysed by Tracer (http://beast.bio.ed.ac.uk/Tracer). The trees were visualized by FigTree (http://tree.bio.ed.ac.uk/software/figtree/) and edited on CorelDraw (http://tree.bio.ed.ac.uk/software/figtree/). Considering the low resolution of the single-genome region Bayesian analyses for *Trichoderma harzianum* complex species, a *p*-distance analysis was performed, following Chaverri *et al*. [29], by MEGA6 [35] with the intention of differentiating the cryptic species.

### 2.3. Experiments *in vitro*

#### 2.3.1. Trichoderma isolates versus *Leucoagaricus*

The symbiotic fungus *Leucoagaricus* was isolated from the fungal gardens of 1-year-old laboratory colonies of *Atta sexdens rubropilosa*. For the experiment, agar discs (1 cm in diameter) were taken from a *Leucoagaricus* mother culture and aseptically transferred to plates containing a medium modified from Pagnocca *et al.* [36], composed of (gl-1) glucose, NaCl, peptone, malt extract, agar, oat flakes, agar, distilled water up to 1 l and the antibiotic chloramphenicol, and then autoclaved at 120°C at 1.1 atm for 30 min. Cultures were maintained in an incubator (25 ± 2°C) for one month prior to use.

Five isolates of the purported endophytic *Trichoderma* strains (see table 1 and electronic supplementary material, table S1) were used in the *in vitro* experiments to assess the interactions with *Leucoagaricus*. The isolates were grown on potato carrot agar (PCA) [37] for 7 days, and agar discs (1 cm diameter) were taken with a cork borer for use as the inoculum. A disc of each *Trichoderma* isolate was used to inoculate PCA plates, pre-inoculated 30 days previously with 1 cm-diameter discs of *Leucoagaricus,* with discs placed in the centre of the 9 cm-diameter plates. Control plates consisted of a disc containing media only, instead of the *Leucoagaricus*. Daily measurements of radial growth of *Trichoderma* were made for 5 days. Each treatment was replicated five times.

**Table 1.** *Trichoderma* isolates that were reisolated from seedlings of *Eucalyptus grandis* inoculated with different species of *Trichoderma* and without *Trichoderma* (Control). Four plant sections were analysed: the pair of leaves that was inoculated (0), the pair above the leaves that were inoculated (−1), the pair of leaves above those that were inoculated (+1), the pair above the pair +1 (+2) and the bud.

		leaves				
	*Trichoderma* isolates	−1	0	+1	+2	bud
TR 01	*Trichoderm atroviride*	4/5	2/5	2/5	2/5	0/5
TR 05	*Trichoderm koningiopsis*	1/5	4/5	1/5	1/5	0/5
TR 07	*Trichoderm spirale*	2/5	0/5	1/5	1/5	0/5
TR 09	*Trichoderm atroviride*	2/5	5/5	0/5	0/5	0/5
TR 25	*Trichoderm koningiopsis*	4/5	3/5	0/5	0/5	0/5
TR 26	*Trichoderm koningiopsis*	3/5	3/5	2/5	2/5	0/5
TR 28	*Trichoderm koningiopsis*	3/5	5/5	5/5	5/5	0/5
TR 33	*Trichoderm atroviridae*	4/5	4/5	0/5	0/5	0/5
TR 49	*Trichoderm spirale*	4/5	0/5	0/5	0/5	0/5
TR 71	*Trichoderm koningiopsis*	0/5	0/5	0/5	0/5	0/5
C	control	0/5	0/5	0/5	0/5	0/5

#### 2.3.2. Proof of endophytism of *Trichoderma* isolates

Ten isolates of *Trichoderma*—found previously to be associated with *Atta sexdens rubropilosa*—were tested for endophytism in *Eucalyptus grandis* (Myrtaceae). For inoculation, a total of 150 seedlings, approximately 15 cm in height, were acquired from a forestry nursery (Viveiro de Mudas do IEF, Viçosa-MG). The seedlings were inoculated by brushing with spore suspensions (SDW containing 0.01% Tween 80^®^ and spores) on the apical bud and the first two leaves (the latter were marked with water-based correction liquid in order to know which leaves were inoculated). Controls were brushed with SDW containing Tween 80^®^ only. All treated shoots were covered with plastic bags for 48 h, after which the bags were removed and the plants were placed in an irrigated greenhouse. After 30 days, five seedlings of each treatment were analysed for *Trichoderma.* For this, four leaf pairs of each seedling were removed. These leaf pairs were: −1 (below the point of initial inoculation), 0 (point of inoculation), +1 (above the point of initial inoculation) and +2 (second pair of leaves above the point of inoculation and the apical bud). As above, four segments (5 mm^2^) were cut from the middle lamina of each leaf (plus control) and surface-sterilized through sequential immersion in 70% ethanol (1 min), 5% sodium hypochlorite (4 min) and washed three times in SDW. Sterilized leaf pieces were placed on VBA and were incubated at 25 ± 2°C.

#### 2.3.3. Behaviour of leaf-cutting ants in the presence of *Trichoderma*

To verify the effects of *Trichoderma* in nests of *Atta sexdens rubropilosa,* behaviour of ants and the death of colonies, we followed a methodology adapted from Jaccoud *et al*. [38] using 10 queenless mini-colonies of *A. sexdens rubropilosa*. To prepare the mini-colonies, samples of approximately 350 ml of fresh fungal garden, containing ants and brood, were taken from 10 different colonies of *A. sexdens rubropilosa* and placed in plastic pots (350 ml volume), and covered with ventilated lids. Next, 2-cm diameter exit holes were made in the base to allow for the passage of worker ants for foraging. The pots were placed along the edges of trays (30 × 25 × 5 cm), whose sides were treated with talcum powder in order to prevent the escape of ants. These colonies were maintained at 25 ± 2°C, 75 ± 3% RH with a 12 L : 12 D photoperiod, in the laboratory. Colonies had been supplied daily with leaves of *Acalypha wilkesiana* (Euphorbiaceae) (3.5 g colony^−1^) for 7 days prior to the treatment. Then, following a period of 12 h without leaves, the ants were offered rice (10 g colony^−1^) colonized by endophytic *Trichoderma* (mycelium and spores) or free of the fungus (control) for 3 days. Subsequently, ants were offered only *A. wilkesiana*, as above.

Preparation of the *Trichoderma-*contaminated substrate followed the methodology of Jenkins *et al.* [39], with minor adaptations: 100 g of white rice and 80 ml of distilled water were placed in polypropylene bags (20 cm × 30 cm × 0.06 mm). The bags were left for 30 min for the rice to absorb water, with frequent shaking to maintain the homogeneity of the substrate. After that, the bags were autoclaved at 120°C and 1.1 atm for 30 min. Once cool, the bags were transferred to a laminar flow cabinet and each of these was inoculated with five mycelial discs (5 mm diameter) of *Trichoderma*. The control consisted of bags inoculated with discs of PCA only. The bags were sealed and transferred to a bench at 25 ± 2°C for 7 days and turned every 2 days to promote gaseous exchange, and to increase surface contact between the fungus and the substrate, as well as to break up the mycelium and thus ensure homogenization. In preliminary tests, it took 8 days until the treatment was totally colonized by *Trichoderma*, and so ready to be offered to the ants.

Over the experimental period, three records were made: the consumption of leaves (each mini-colony received 3.5 g of leaves/per day that were weighed 24 h later), the weight of fresh waste and the survival of the ant colonies. The latter was measured by counting the number of dead ants in the waste. All records were taken daily.

### 2.4. Statistical analyses

To verify if the frequency of endophytic *Trichoderma* differed between the vegetative material that was being carried or that had been rejected by *A. sexdens rubropilosa*, tests of independence were conducted, incorporating *G*-tests [40]. For the *in vitro* experiment, comparisons were made of the mean radial growth of *Trichoderma* isolates in the presence or the absence of *Leucoagaricus* using two-tailed *t-*tests. Data from the third day of growth were used as these presented the largest differences*.* For the experiments of the endophytic ability of *Trichoderma*, only the presence or the absence of the isolates in each selected leaf part of *E. grandis* were recorded*.* Finally, the analyses of behaviour of mini-colonies between control and treatments were tested using Mann–Whitney *U*-tests. The survival curves were obtained using Kaplan–Meier survival distributions in the R software [41].

## 3. Results

### 3.1. *Trichoderma* in leaf material from *Atta sexdens rubropilosa* nests

**Figure 1.**
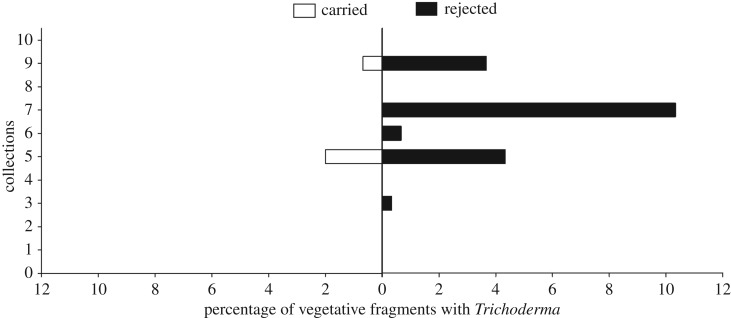
Frequencies of occurrence of *Trichoderma* in leaf pieces that were being carried towards the nest by *Atta sexdens rubropilosa* workers (‘carried’ on the left) or that had been rejected and were found outside the entrances (‘rejected’ on the right). Shown are percentages of pieces with *Trichoderma* found in ten weekly collections.

Of the total of 6000 leaf pieces collected from six nests of *A. sexdens rubropilosa*, we found 66 *Trichoderma* isolates (1.1%). Of these isolates, eight (12.12%) were in material that was being carried to the nest and 58 (87.88%) were adjacent to the nest entrance in the rejected material (figure 1). This represented a significant difference (value of *G* = 73.02, greater than $\chi_{\text{d.f. = 1}}^{2}=3.84$ for *p* = 0.05). In the collections of leaves from plants around the foraging areas of the six nests, *Trichoderma* was isolated only from old leaves of two plants in one of the forest fragments (Mata do Paraíso).

### 3.2. Molecular identification

**Figure 2.**
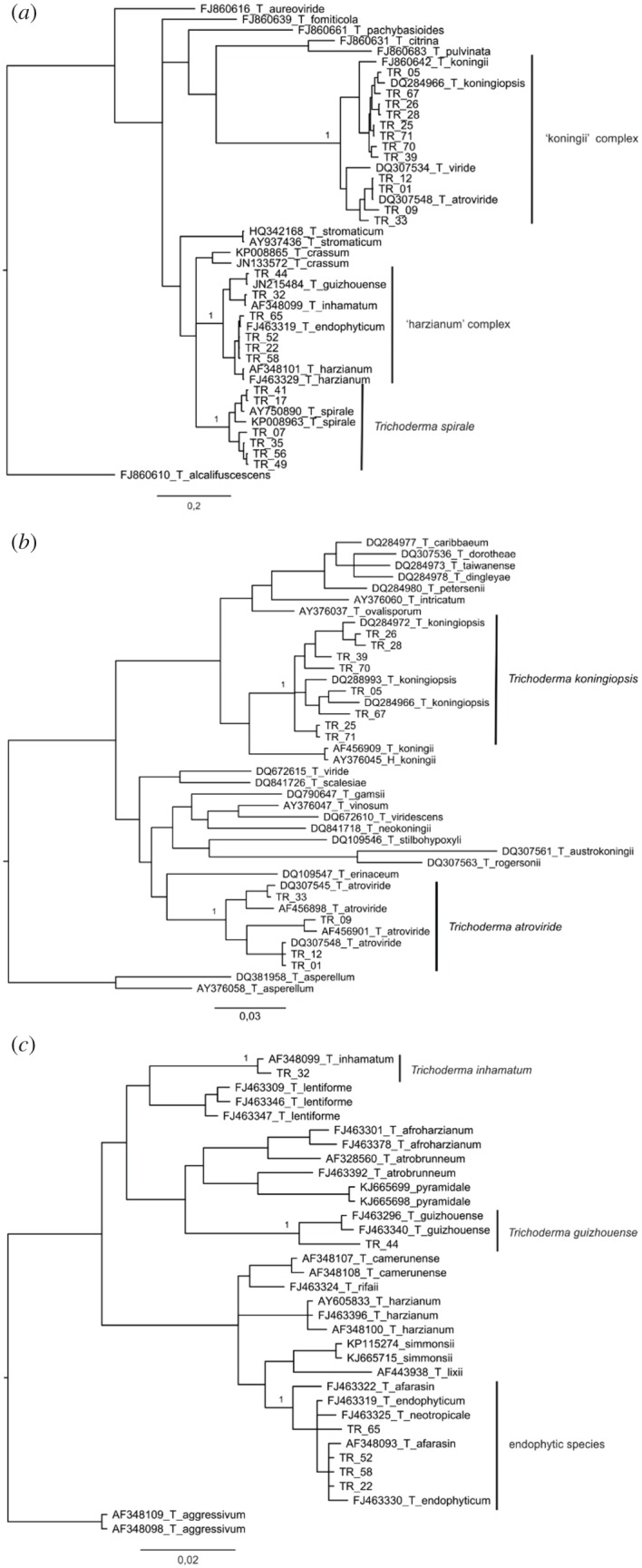
(*a,b*) Phylogenetic tree including species from the *Trichoderma koningii* complex. The resulting tree was obtained from Bayesian inference of TEF1. Strains belonging to *Trichoderma atroviride* and *T. koningiopsis* were identified by the analysis. (*c*) Species of the *Trichoderma harzianum* complex compared by a resulting tree obtained from Bayesian inference of TEF1. The analysis was not capable of differentiating the species *T. afarasin*, *T. endophyticum* and *T. neotropicale*. Some strains were identified as *T. inhamatum* and *T. guishouense*.

Six *Trichoderma* species were identified (figure 2*a–c* and electronic supplementary material, table S2). The first Bayesian analysis identified six strains as *T. spirale*. Other strains were grouped in two different species complexes: 12 in the *T. koningii* complex and six in the *T. harzianum* complex. The Bayesian analysis performed with *koningii*-complex strains showed eight strains belonging to *T. koningiopsis* and four strains belonging to *T. atroviride*. The Bayesian analysis performed with *harzianum*-complex strains failed on species identification, thus the *p*-distance analysis identified four strains as *T. endophyticum*, one strain as *T. guizhouense* and one as *T. inhamatum*.

### 3.3. Experiments *in vitro*

#### 3.3.1. *Trichoderma* isolates versus *Leucoagaricus*

**Figure 3.**
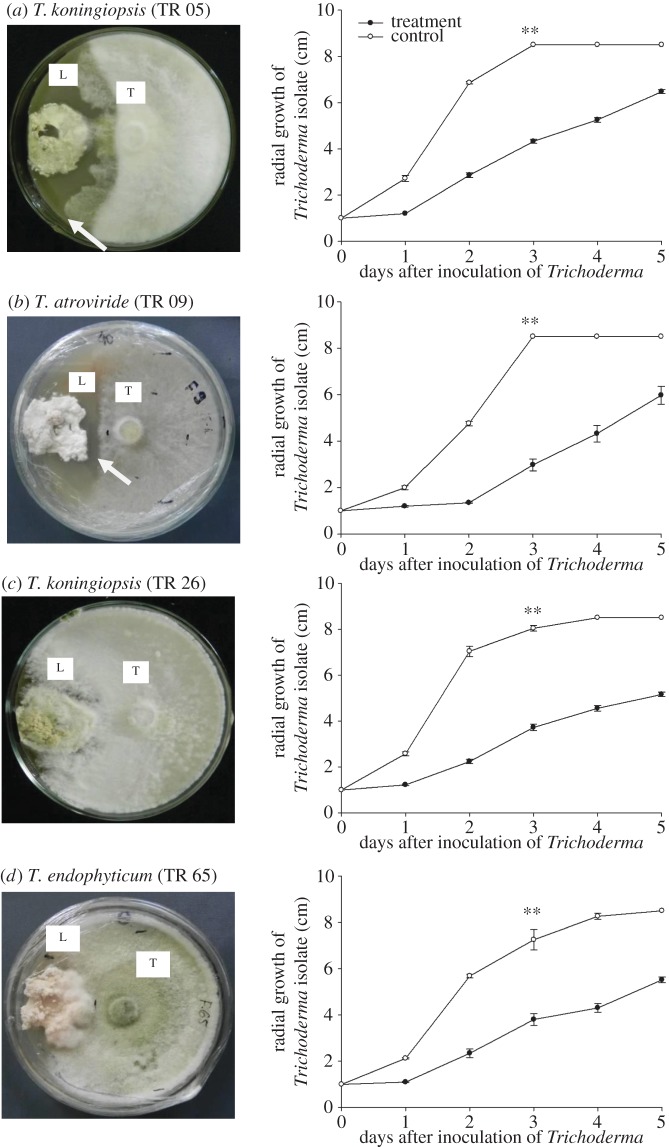
(*a*--*d*) Mean radial growth of *Trichoderma* isolates in paired cultures with *Leucoagaricus*, after 30 days of incubation. Two-tailed *t*-tests were performed comparing growth by the third day between treatment and control. L, *Leucoagaricus*; T, *Trichoderma*. Arrows indicate zones of inhibition. ** denotes significant differences (*t*-tests, *p* ≤ 0.001) between treatment and control on day 3.

In all interactions between the mutualistic fungus *Leucoagaricus* and isolates of *Trichoderma*, the growth of *Trichoderma* isolates was reduced when compared with the controls at day 3: isolate 5: *t*_1,8_ = −26.80, *p* < 0.0001; isolate 9: *t*_1,8_ = −19.92, *p* < 0.0001; isolate 26: *t*_1,8_ = −31.39 *p* < 0.0001; isolate 32: *t*_1,8_ = −2.65, *p* = 0.0164; and isolate 65: *t*_1,8_ = −4.11, *p* = 0.0017 (figure 3).

In some of the interactions between *Leucoagaricus* and *Trichoderma*, an inhibition zone, or halo, was observed around the *Leucoagaricus* colonies, indicating a defence reaction on the part of *Leucoagaricus* against certain *Trichoderma* strains (figure 3*a,b*). However, by day 5 most of the isolates had overcome these defences and had colonized the symbiont (figure 3*c,d*).

#### 3.3.2. Testing the endophytism of *Trichoderma* isolates

Ten isolates of *Trichoderma* were tested for endophytism in eucalyptus seedlings. None of the isolates of *Trichoderma* were reisolated from the bud (electronic supplementary material, table S2). Four isolates (*Trichoderma koningiopsis*, TR 05, TR 26, TR 28 (2) and *T. atroviride,* TR 01) were reisolated from the area of inoculation (0), above it (+1, +2) and below (−1) (electronic supplementary material, table S2). For three isolates (*T. atroviride*, TR 09; *T. koningiopsis*, TR 25; *T. atroviride*, TR 33), the reisolation occurred in the inoculated area (0) and below (−1) (electronic supplementary material, table S2). Two of the isolates (*T. spirale,* TR 07; *T. spirale,* TR 49) were not reisolated from the inoculated area but were present in leaves below it (−1); while isolate TR 07 was also isolated from the leaves above (+1; +2). Isolate *T. koningiopsis*, TR 71 was not recovered from any of the tissues (table 1).

#### 3.3.3. Behaviour of leaf-cutting ants in the presence of *Trichoderma*

**Figure 4.**
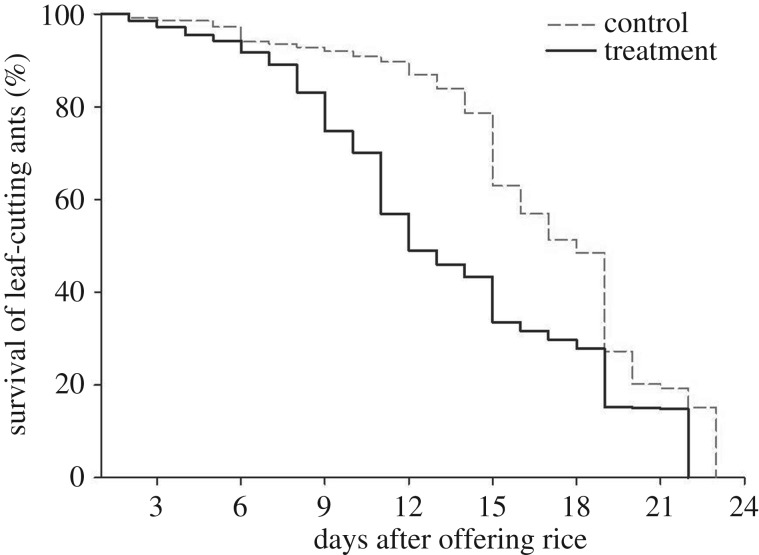
Survival curves of workers of *Atta sexdens rubropilosa* of queenless colonies to which were offered rice containing *Trichoderma* and without (control). The mortality of the leaf-cutting ant was assessed daily. The death of the colony was recorded when all workers were dead.

All mini-colonies took rice, independently of its colonization or not by *Trichoderma*, and incorporated it in the fungal garden (while there may have been spores on the surface of the rice grains, it did not affect this initial behaviour). In comparing the colonies from the control and the treatments, there were no significant differences in weights of leaves taken (*U* = 2352.5, d.f. = 1, *p* = 0.37) or of the waste (*U* = 2497.0 d.f. = 1, *p* = 0.085). However, comparisons of survival times showed that ants from the controls survived considerably longer than those from the *Trichoderma-*treated colonies (median of 17 ± 0.0373 days versus 11 ± 0.0801 days *log-rank* statistic *=* 3922, 975; *p* < 0.001; figure 4).

## 4. Discussion

Endophytic *Trichoderma* was isolated more frequently from material rejected from the ant colonies than from that being carried by workers of *A. sexdens rubropilosa* (figure 1). This result is very similar to that for *Trichoderma*-containing material rejected by another leaf-cutter, *A. laevigata* [22]. Based on these field results, we believe that *Trichoderma*—as a potential antagonist of *Leucoagaricus* [23,42,43]—poses a direct threat to the fungal garden and thereby, indirectly, to the ant colony. Thus, once detected within leaf material during quality-control activities within the nest, it is rejected from the nest [44]. In addition, previous field observations show that *Atta* ants adopt a strict pattern of disposal and that rejected leaf material is not scattered randomly around the nest entrances, or heaped in middens, but dropped on the downslopes above or below nest entrances and, in fact, that some of these purported ‘entrances’ may function solely for the disposal of rejected leaves [22]. Although the incidence of *Trichoderma* appears to be low, it is important to re-emphasize that the main finding of this experiment is the significantly higher incidence of this fungus in the rejected leaves compared with those that were carried (indicating a threat recognition by the ants, as has been discussed). Identification of which plant species harbour this endophyte was not feasible in the present study due to the complexity of the plant community and the nature of the methods that would be required to track individual leaf fragments back to a given species. We hypothesize that leaf endophytism of *Trichoderma* is specific to certain species of host plants such that it may be an important component of the defences that the given species employs, with higher incidences in these species than the global incidence we report. While many aspects of such interactions are discussed in papers such as that of Heil & McKey [45], we were not able to find in the literature studies that report incidences of a given defence mechanism of plants in communities such as the one under study here. We suspect that the ‘Trojan Horse’ mutualism we propose may not be sufficiently common as to structure plant communities, but may be important rather: (i) in determining the success of a given species or individual that engages in such a mutualism; (ii) as a further example of the capabilities of the ants in defending their colonies and (iii) as a route towards managing the impact of these pests in agriculture and silviculture. It is clear that this subject requires further attention in the form of field studies focused on identifying the plants involved.

It is possible, therefore, that certain plants may be using endophytic fungi, such as *Trichoderma*, as bodyguards against leaf-cutting ants because of the array of defence mechanisms—including enzymatic and chemical weapons—that they possess [42,43]. This hypothesis is also based on existing evidence that *Trichoderma* is highly antagonistic to members of the Agaricaceae family, which includes the ant symbiont fungus (*Leucoagaricus*). For example, Ortiz & Orduz [46] reported that certain strains of *Trichoderma* inhibit the mycelial growth of *Attamyces* sp. (i.e. *Leucoagaricus*), while various *Trichoderma* species can be important constraints and major pests in the production of commercial crops of *Agaricus* and *Pleurotus* mushrooms [47–49]. In these systems, *Trichoderma* can compete for substrates [50]; or produce antifungal compounds which inhibit growth and fruiting of the mushrooms [51]; or, secrete cell-wall-degrading enzymes which damage the crop [52]. Nevertheless, the conclusion from the present survey is that *Trichoderma* is not a common resident in leaves of tropical trees, since, of the 3000 cut leaves sampled, only around 2% contained the fungus. It could be conjectured, of course, that ants avoid those tree species, or individual trees, which may harbour high levels of endophytic *Trichoderma*. This would provide a significant competitive edge to those trees that do harbour *Trichoderma*. However, in a study screening for endophytes of forest trees—which involved random sampling of leaves from around *Atta* nests—*Trichoderma* isolates were recorded infrequently [22]. Although there are increasing reports of *Trichoderma* as a leaf endophyte [22,25,53,54], previous and ongoing evidence indicates that it has a stronger predilection for woody tissues [24,55–61]; and, moreover, that there is a highly specialized clade that has exploited the endophytic niche, especially in tree hosts [29,62].

Previous studies of leaf-cutting ants have shown that these insects adopt several specific strategies during the collection and processing of leaves [63,64]. In general, they choose leaves based on leaf age [65,66], moisture content [67], leaf toughness [68], secondary toxic components [69], nitrogen content [70] and vegetative material that is unsuitable for the mutualistic fungus [71,72]. More recently, it has been suggested that endophytic fungi should also be taken into account since there is evidence that the leaf-cutting ants spend more time processing and screening leaves containing certain fungal genera [73]. However, studies of the interaction between leaf-cutting ants and plants harbouring endophytic fungi are still at an early stage [74–76].

According to Knapp *et al*. [72] and North *et al*. [77], leaf-cutting ants may be capable of detecting changes in and threats to the fungal garden by responding to semiochemicals produced by the symbiont. Based on this information, our results may indicate rejection of leaves because of the presence of *Trichoderma* due to the potential adverse effects on *Leucoagaricus*. As shown in the dual culture experiment, most of the isolates eventually overcame and colonized the symbiont, despite the initial defence response.

Given the above, the use of endophytic *Trichoderma* as a bodyguard could be considered as a management strategy to protect crops from leaf-cutting ants. There is increasing evidence, for example, that endophytic fungi help to protect plants against herbivorous insects—or herbivores in general—by producing toxins with insecticidal activity [78–81], although most studies have involved grass hosts and the endophytic genus *Neotyphodium* (Hypocreales: Clavicipitaceae) [82–86]. According to these studies, this protection is related to the mode of transmission of *Neotyphodium,* which in grasses is exclusively vertical, within the seeds. Vertical transmission can promote the formation and maintenance of mutualisms, although there are prominent examples of mutualisms without vertical transmission, such as in mycorrhizal symbiosis [87]. Thus, the possibility of plants using *Trichoderma* endophytes as bodyguards against leaf-cutting ants would be associated with more indirect effects by targeting the *Leucoagaricus* symbiont rather than the ants *per se*.

In the *in vitro* experiments, we demonstrated that *Leucoagaricus* has some defence against *Trichoderma;* it is possible to see in figure 3*b* that the inhibition halo has remained. However, for the other isolates, after the fifth day, *Trichoderma* overgrew and colonized *Leucogaricus* (figure 3*c,d*). According to Silva *et al*. [88], the growth of *Leucoagaricus* is negatively affected by *Trichoderma harzianum*, while Van Bael *et al*. [73] showed that the mutualistic fungus can inhibit the growth of the endophytic fungus *Glomerella cingulata* via diffusible antibiotics rather than hyphal contact; although it is difficult to see the relevance of this in the ant–mutualist system because this fungus—essentially, plant pathogenic in habit—would not present a threat to either component. Here, we found that *Leucoagaricus* has a chemical-defensive response to all of the isolates of *Trichoderma* tested, so it is not just dependent on the physical protection of the leaf-cutting ants during ‘weeding’ of the fungal garden. In experiments where the plant material was pretreated with systemic fungicide, the ants initially incorporated the material into the nest and the rejection behaviour was only observed some 10 h later [44]. We interpret that this delayed rejection is the result of a screening or selection process where the threat from the fungicide is perceived, either by the quality-control system operated by the ants or, possibly, by semiochemicals from the mutualistic fungus itself.

In the test for endophytism—with the ultimate goal of developing a biological control product that could be used to protect crops—the results showed that there was systemic movement of all the *Trichoderma* strains (table 1). In general, however, endophytism was relatively weak and this could be related to host specificity issues in endophytic *Trichoderma*, although the relation between plants and endophytic fungi is considered to be less specialized than with other symbioses [89]. Nevertheless, isolate screening could still be a critical factor in the selection of potential bodyguards and work is in progress to collect endophytic *Trichoderma* isolates from endemic members of the Myrtaceae. It has been demonstrated, for example, that in *Theobroma cacao*, selected isolates of *Trichoderma* from wild species of *Theobroma*, were able to colonize the cacao tissues in the area of inoculation and move to the roots, stems and leaves, as well as to the growing points [59]. However, the protocol adopted in the latter study involved inoculating very young plants (newly germinated cacao beans), rather than established seedlings, and the use of older plant tissues in our study may have resulted in reduced endophytic colonization.

Our study of the effect of *Trichoderma* on the survival and behaviour of the *Atta* workers showed no effects on the collecting pattern (the weight of harvested leaves). We expected that the presence of *Trichoderma* would cause colonies to harvest more material but this did not happen. The lack of a difference could be attributed to the ants not perceiving the presence of the fungus or to communication between the ants and *Leucoagaricus* being affected in the absence of the queen. The lack of a difference in the weight of waste could be ascribed to an absence of infection in the ants' mutualist fungus because of hygienic behaviour of the ants. Currie & Stuart [90] highlighted the capacity of ants to remove microbial pathogens from the fungus garden, and this information supports our results, since we did not observe differences between the treatment and the control. There was a reduction in ant survival time in the presence of *Trichoderma* (figure 3), and it is possible that mortality is associated with insecticidal activity of the fungus, as has been reported for formulations of *Trichoderma harzianum* against aphids [91]. These authors also suggested that the presence of degrading enzymes (chitinases, proteases) might also help to facilitate the entry of insect toxins. Interpretation of our results is naturally limited by considerations of experimental design. Thus, the colonies used here were queenless and the absence of the queen may have increased worker mortality. It could also decrease waste accumulation (against a possible increase due to *Trichoderma* contamination) as the lack of a queen may disrupt internal colony tasks, such as fungal garden maintenance [92]. The use of whole colonies would be the ideal; however, mini-colonies are still considered to be an adequate tool when there are no nests available for laboratory experiments.

In conclusion, we found *Trichoderma* as an endophyte in both carried and rejected vegetative material associated with *A. sexdens rubropilosa*, but significantly more (*p* = 0.05) in the rejected material. This work opens up the possibility of exploiting these fungi as bodyguards to protect crop plants against leaf-cutting ants. However, further work is necessary to confirm this relationship and, in particular, to investigate the processing of material by leaf-cutting ants below-ground. Regarding the *in vitro* experiments, *Leucoagaricus* was able to inhibit the negative effect of some of the *Trichoderma* isolates for some days, which would possibly be sufficient to communicate to its ant mutualists that the material is potentially harmful. Endophytism of most of the *Trichoderma* isolates was inconsistent; possibly due to the inoculation methodology employed, with relatively low levels of recovery (0–40%) in seedlings of *E. grandis*, and could be classed as weakly to moderately endophytic. Nevertheless, one isolate of *T. koningiopsis* demonstrated a high level of systemic ability and was isolated from over 70% of the tissues, but not from the apical meristem. The ‘perfect’ endophyte would move with and colonize the meristematic tissues, thus affording protection to the whole plant.

The bodyguard hypothesis is currently being tested in the greenhouse and in the field with the long-term aim of developing a strategy for the management of leaf-cutting ants, with particular reference to eucalyptus, a major plantation tree in Minas Gerais, and within Brazil, in general.

## Supplementary Material

Table S1; Table S2

## Data accessibility

DNA sequences: GenBank accession no. KT61055–KT61078. TreeBASE accession number S16556 and S19555 (http://www.treebase.org/). Other data are available in http://dx.doi.org/10.5061/dryad.0164h [93].
