# Supplementary material for: Recognition of endophytic Trichoderma species by leaf-cutting ants and their potential in a Trojan-horse management strategy
Source: R Soc Open Sci. 2017 Apr 5;4(4):160628. doi: 10.1098/rsos.160628 (PMC5414240; doi:10.1098/rsos.160628)
Supplement: Table S1; Table S2 [file rsos160628supp1.docx]

Supplementary material

Table S1. Origins of *Trichoderma* isolated from leaves cut by *Atta sexdens rubropilosa* in Viçosa-MG/Brazil. Rejected leaves were considered to be those scattered by the ants above or at the side of the nest entrances. Carried leaves were taken from ants as they were being transported to the nest.

|  | **Species** | | **Material** | | **Geographic/origin** | | **GenBank numbers** |
| --- | --- | --- | --- | --- | --- | --- | --- |
| TR 01 | *Trichoderma atroviride* | Rejected leaves | | Mata do Seu Nico/Viçosa-MG | | KT619061 | |
| TR 05 | *T. koningiopsis* | Rejected leaves | | Mata do Seu Nico/Viçosa-MG | | KT619057 | |
| TR 07 | *T. spirale* | Rejected leaves | | Mata do Seu Nico/Viçosa-MG | | KT619065 | |
| TR 09 | *T. atroviride* | Rejected leaves | | Mata do Seu Nico/Viçosa-MG | | KT619067 | |
| TR 12 | *T. atroviride* | Rejected leaves | | Mata do Paraíso/Viçosa-MG | | KT619055 | |
| TR 17 | *T. spirale* | Rejected leaves | | Mata do Paraíso /Viçosa-MG | | KT619064 | |
| TR 22 | *T. endophyticum* | Rejected leaves | | Mata do Paraíso /Viçosa-MG | | KT619074 | |
| TR 25 | *T. koningiopsis* | Rejected leaves | | Mata do Paraíso/Viçosa-MG | | KT619072 | |
| TR 26 | *T. koningiopsis* | Rejected leaves | | Mata do Seu Nico/Viçosa-MG | | KT619060 | |
| TR 28 | *T. koningiopsis* | Rejected leaves | | Mata do Seu Nico/Viçosa-MG | | KT619078 | |
| TR 32 | *T. inhamatum* | Rejected leaves | | Mata do Seu Nico/Viçosa-MG | | KT619076 | |
| TR 33 | *T. atroviride* | Carried leaves | | Mata do Paraíso/Viçosa-MG | | KT619073 | |
| TR 35 | *T. spirale* | Rejected leaves | | Mata do Seu Nico/Viçosa-MG | | KT619062 | |
| TR 39 | *T. koningiopsis* | Rejected leaves | | Mata do Seu Nico/Viçosa-MG | | KT619070 | |
| TR 41 | *T. spirale* | Rejected leaves | | Mata do Seu Nico/Viçosa-MG | | KT619063 | |
| TR 44 | *T. guizhouense* | Carried leaves | | Mata do Paraíso/Viçosa-MG | | KT619059 | |
| TR 49 | *T. spirale* | Rejected leaves | | Mata do Seu Nico/Viçosa-MG | | KT619069 | |
| TR 52 | *T. endophyticum* | Rejected leaves | | Mata do Paraíso/Viçosa-MG | | KT619058 | |
| TR 56 | *T. spirale* | Rejected leaves | | Mata do Seu Nico/Viçosa-MG | | KT619071 | |
| TR 58 | *T. endophyticum* | Rejected leaves | | Mata do Paraíso/Viçosa-MG | | KT619066 | |
| TR 65 | *T. endophyticum* | Rejected leaves | | Mata do Seu Nico/Viçosa-MG | | KT619056 | |
| TR 67 | *T. koningiopsis* | Carried leaves | | Mata do Paraíso/Viçosa-MG | | KT619068 | |
| TR 70 | *T. koningiopsis* | Rejected leaves | | Mata do Paraíso/Viçosa-MG | | KT619075 | |
| TR 71 | *T. koningiopsis* | Carried leaves | | Mata do Seu Nico/Viçosa-MG | | KT619077 | |

Table S2. Estimates of evolutionary divergence (p-distance) over sequence pairs between species in the *T. harzianum* complex. The analyss is based on number of base differences per site from averaging over the entire elongation factor (TEF1) sequence. The analysis involved 184 nucleotide sequences. All ambiguous positions were removed for each sequence pair. There were a total of 486 positions in the final dataset.

| **Species 1** | **Species 2** | **Dist** | **Std. Err** |
| --- | --- | --- | --- |
| *afarasin* | *endophyticum* | 0,048 | 0,007 |
| *afarasin* | *neotropicale* | 0,058 | 0,008 |
| *endophyticum* | *neotropicale* | 0,045 | 0,006 |
| *afarasin* | *lentiforme* | 0,108 | 0,013 |
| *endophyticum* | *lentiforme* | 0,105 | 0,013 |
| *neotropicale* | *lentiforme* | 0,123 | 0,015 |
| *afarasin* | TR_22_52_58_65 | 0,039 | 0,006 |
| *endophyticum* | TR_22_52_58_65 | 0,021 | 0,004 |
| *neotropicale* | TR_22_52_58_65 | 0,042 | 0,007 |
| *lentiforme* | TR_22_52_58_65 | 0,095 | 0,013 |
| *afarasin* | *simmonsii* | 0,055 | 0,008 |
| *endophyticum* | *simmonsii* | 0,045 | 0,008 |
| *neotropicale* | *simmonsii* | 0,064 | 0,010 |
| *lentiforme* | *simmonsii* | 0,095 | 0,013 |
| TR_22_52_58_65 | *simmonsii* | 0,033 | 0,008 |
| *afarasin* | *camerunense* | 0,054 | 0,008 |
| *endophyticum* | *camerunense* | 0,044 | 0,008 |
| *neotropicale* | *camerunense* | 0,070 | 0,011 |
| *lentiforme* | *camerunense* | 0,093 | 0,013 |
| TR_22_52_58_65 | *camerunense* | 0,028 | 0,007 |
| *simmonsii* | *camerunense* | 0,031 | 0,008 |
| *afarasin* | *rifaii* | 0,054 | 0,007 |
| *endophyticum* | *rifaii* | 0,045 | 0,007 |
| *neotropicale* | *rifaii* | 0,070 | 0,010 |
| *lentiforme* | *rifaii* | 0,086 | 0,011 |
| TR_22_52_58_65 | *rifaii* | 0,028 | 0,007 |
| *simmonsii* | *rifaii* | 0,033 | 0,008 |
| *camerunense* | *rifaii* | 0,024 | 0,007 |
| *afarasin* | *harzianum* | 0,061 | 0,008 |
| *endophyticum* | *harzianum* | 0,053 | 0,009 |
| *neotropicale* | *harzianum* | 0,081 | 0,012 |
| *lentiforme* | *harzianum* | 0,105 | 0,013 |
| TR_22_52_58_65 | *harzianum* | 0,035 | 0,008 |
| *simmonsii* | *harzianum* | 0,047 | 0,010 |
| *camerunense* | *harzianum* | 0,034 | 0,008 |
| *rifaii* | *harzianum* | 0,036 | 0,008 |
| *afarasin* | *lixii* | 0,060 | 0,011 |
| *endophyticum* | *lixii* | 0,052 | 0,011 |
| *neotropicale* | *lixii* | 0,061 | 0,011 |
| *lentiforme* | *lixii* | 0,080 | 0,014 |
| *TR_22_52_58_65* | *lixii* | 0,045 | 0,012 |
| *simmonsii* | *lixii* | 0,032 | 0,010 |
| *camerunense* | *lixii* | 0,047 | 0,012 |
| *rifaii* | *lixii* | 0,042 | 0,011 |
| *harzianum* | *lixii* | 0,064 | 0,013 |
| *afarasin* | *afroharzianum* | 0,112 | 0,013 |
| *endophyticum* | *afroharzianum* | 0,112 | 0,013 |
| *neotropicale* | *afroharzianum* | 0,133 | 0,015 |
| *lentiforme* | *afroharzianum* | 0,083 | 0,011 |
| TR_22_52_58_65 | *afroharzianum* | 0,096 | 0,013 |
| *simmonsii* | *afroharzianum* | 0,096 | 0,014 |
| *camerunense* | *afroharzianum* | 0,090 | 0,013 |
| **Species 1** | **Species 2** | **Dist** | **Std. Err** |
| *rifaii* | *afroharzianum* | 0,084 | 0,012 |
| *harzianum* | *afroharzianum* | 0,098 | 0,013 |
| *lixii* | *afroharzianum* | 0,077 | 0,015 |
| *afarasin* | *atrobrunneum* | 0,112 | 0,012 |
| *endophyticum* | *atrobrunneum* | 0,106 | 0,013 |
| *neotropicale* | *atrobrunneum* | 0,123 | 0,014 |
| *lentiforme* | *atrobrunneum* | 0,084 | 0,011 |
| TR_22_52_58_65 | *atrobrunneum* | 0,093 | 0,013 |
| *simmonsii* | *atrobrunneum* | 0,090 | 0,013 |
| *camerunense* | *atrobrunneum* | 0,087 | 0,012 |
| *rifaii* | *atrobrunneum* | 0,084 | 0,011 |
| *harzianum* | *atrobrunneum* | 0,101 | 0,013 |
| *lixii* | *atrobrunneum* | 0,076 | 0,015 |
| *afroharzianum* | *atrobrunneum* | 0,049 | 0,009 |
| *afarasin* | *pyramidale* | 0,106 | 0,013 |
| *endophyticum* | *pyramidale* | 0,094 | 0,012 |
| *neotropicale* | *pyramidale* | 0,114 | 0,014 |
| *lentiforme* | *pyramidale* | 0,070 | 0,010 |
| TR_22_52_58_65 | *pyramidale* | 0,082 | 0,012 |
| *simmonsii* | *pyramidale* | 0,078 | 0,013 |
| *camerunense* | *pyramidale* | 0,076 | 0,012 |
| *rifaii* | *pyramidale* | 0,073 | 0,011 |
| *harzianum* | *pyramidale* | 0,090 | 0,013 |
| *lixii* | *pyramidale* | 0,058 | 0,014 |
| *afroharzianum* | *pyramidale* | 0,060 | 0,010 |
| *atrobrunneum* | *pyramidale* | 0,034 | 0,007 |
| *afarasin* | *guizhouense* | 0,119 | 0,013 |
| *endophyticum* | *guizhouense* | 0,109 | 0,013 |
| *neotropicale* | *guizhouense* | 0,135 | 0,015 |
| *lentiforme* | *guizhouense* | 0,081 | 0,011 |
| TR_22_52_58_65 | *guizhouense* | 0,096 | 0,013 |
| *simmonsii* | *guizhouense* | 0,102 | 0,013 |
| *camerunense* | *guizhouense* | 0,089 | 0,013 |
| *rifaii* | *guizhouense* | 0,088 | 0,012 |
| *harzianum* | *guizhouense* | 0,101 | 0,013 |
| *lixii* | *guizhouense* | 0,090 | 0,016 |
| *afroharzianum* | *guizhouense* | 0,064 | 0,010 |
| *atrobrunneum* | *guizhouense* | 0,064 | 0,010 |
| *pyramidale* | *guizhouense* | 0,050 | 0,009 |
| *afarasin* | TR_44 | 0,123 | 0,014 |
| *endophyticum* | TR_44 | 0,115 | 0,014 |
| *neotropicale* | TR_44 | 0,143 | 0,016 |
| *lentiforme* | TR_44 | 0,088 | 0,013 |
| TR_22_52_58_65 | TR_44 | 0,101 | 0,014 |
| *simmonsii* | TR_44 | 0,111 | 0,015 |
| *camerunense* | TR_44 | 0,095 | 0,013 |
| *rifaii* | TR_44 | 0,091 | 0,012 |
| *harzianum* | TR_44 | 0,109 | 0,014 |
| *lixii* | TR_44 | 0,103 | 0,018 |
| *afroharzianum* | TR_44 | 0,071 | 0,011 |
| *atrobrunneum* | TR_44 | 0,071 | 0,011 |
| *pyramidale* | TR_44 | 0,056 | 0,011 |
| *guizhouense* | TR_44 | 0,017 | 0,004 |
| **Species 1** | **Species 2** | **Dist** | **Std. Err** |
| *afarasin* | *inhamatum* | 0,115 | 0,013 |
| *endophyticum* | *inhamatum* | 0,104 | 0,013 |
| *neotropicale* | *inhamatum* | 0,129 | 0,015 |
| *lentiforme* | *inhamatum* | 0,073 | 0,011 |
| TR_22_52_58_65 | *inhamatum* | 0,088 | 0,013 |
| *simmonsii* | *inhamatum* | 0,082 | 0,013 |
| *camerunense* | *inhamatum* | 0,084 | 0,012 |
| *rifaii* | *inhamatum* | 0,080 | 0,012 |
| *harzianum* | *inhamatum* | 0,098 | 0,013 |
| *lixii* | *inhamatum* | 0,070 | 0,015 |
| *afroharzianum* | *inhamatum* | 0,082 | 0,012 |
| *atrobrunneum* | *inhamatum* | 0,083 | 0,012 |
| *pyramidale* | *inhamatum* | 0,072 | 0,012 |
| *guizhouense* | *inhamatum* | 0,073 | 0,011 |
| TR_44 | *inhamatum* | 0,081 | 0,013 |
| *afarasin* | TR_32 | 0,117 | 0,013 |
| *endophyticum* | TR_32 | 0,106 | 0,013 |
| *neotropicale* | TR_32 | 0,131 | 0,015 |
| *lentiforme* | TR_32 | 0,076 | 0,011 |
| TR_22_52_58_65 | TR_32 | 0,090 | 0,013 |
| *simmonsii* | TR_32 | 0,085 | 0,013 |
| *camerunense* | TR_32 | 0,086 | 0,013 |
| *rifaii* | TR_32 | 0,082 | 0,012 |
| *harzianum* | TR_32 | 0,100 | 0,013 |
| *lixii* | TR_32 | 0,074 | 0,015 |
| *afroharzianum* | TR_32 | 0,084 | 0,012 |
| *atrobrunneum* | TR_32 | 0,085 | 0,012 |
| *pyramidale* | TR_32 | 0,075 | 0,012 |
| *guizhouense* | TR_32 | 0,076 | 0,011 |
| TR_44 | TR_32 | 0,084 | 0,013 |
| *inhamatum* | TR_32 | 0,002 | 0,002 |
